# Encountering abuse in health care; lifetime experiences in postnatal women - a qualitative study

**DOI:** 10.1186/1471-2393-13-74

**Published:** 2013-03-22

**Authors:** Anne-Mette Schroll, Hanne Kjærgaard, Julie Midtgaard

**Affiliations:** 1 Rigshospitalet, Copenhagen University Hospital, Center for Foetal Medicine and Pregnancy (4002), Blegdamsvej 9, Copenhagen Ø, DK-2100, Denmark; 2 Rigshospitalet, Copenhagen University Hospital, The Research Unit Women’s and Children’s Health (7821), Blegdamsvej 9, Copenhagen Ø, DK-2100, Denmark; 3 Rigshospitalet, Copenhagen University Hospital, Center for Health Care Research (UCSF) (9701), Blegdamsvej 9, Copenhagen Ø, DK-2100, Denmark; 4 Department of Clinical Medicine, University of Copenhagen, Faculty of Health and Medical Sciences, Blegdamsvej 3, Copenhagen Ø, DK-2100, Denmark

**Keywords:** Abuse in health care, Pregnancy, Childbirth, Dehumanization, Empathy

## Abstract

**Background:**

Abuse in health care (AHC) has been associated with potential severe health consequences, and has further been related to maternal morbidity and mortality in childbirth. To improve our understanding of what qualifies as AHC and to support and optimise the health of women with these experiences, the objective of this study was to describe how women, who had previously endured AHC, gave meaning to and managed their experience during pregnancy, childbirth, and in the early postnatal period.

**Method:**

Women, who had reported substantial suffering as a result of a previous experience of abuse within the healthcare system, were purposefully selected from a Danish sample of a multinational cohort study on negative life events among pregnant women (the BIDENS Study). Eleven women were interviewed individually by means of a semi-structured interview guide. Transcripts of the interviews were analysed by means of qualitative systematic text condensation analysis.

**Results:**

Four categories were identified to describe the women’s experience of AHC and its consequences on pregnancy and childbirth: abusive acts of unintentional harm, dehumanization, bodily remembrance, and finding the strength to move on. Abuse in health care may have profound consequences on the reproductive lives of the women, among others affecting sexuality, the desire to have children and the expectations of mode of delivery. However, the women described constructive ways to manage the experience, to which healthcare professionals could also contribute significantly.

**Conclusions:**

Regardless of whether AHC is experienced in childhood or adulthood, it can influence the lives of women during pregnancy and childbirth. By recognising the potential existence of AHC, healthcare professionals have a unique opportunity to support women who have experienced AHC.

## Background

Between 13–28% of women attending gynecological clinics in the northern European countries have experienced abuse within the healthcare system (AHC) [1,2]. AHC is defined by patients’ subjective experiences and is characterized by devoid of care, suffering and loss of value as a human being [3]. AHC has been associated with potential severe health consequences such as post-traumatic stress symptoms, sleeping problems, and poor self-rated health [2]. Further, AHC has been related to maternal morbidity and mortality in childbirth [4,5], and it has been emphasized, that disrespect and abuse in childbirth constitute important causes of suffering and human right violation for women in many countries [4,6]. No agreed definition of AHC [5] exists, but d’Oliveira et al. described four different types of AHC: neglect, verbal-, physical- and sexual abuse. Other forms of AHC, such as structural violence, have also been emphasised [5]. However, only few studies have identified AHC and its consequences among a western population of women, and more studies are needed to describe the character and the meaning of the events perceived by patients as AHC [7].

According to the UN, reproductive health is a crucial part of general health, a central feature of human development, setting the stage for health beyond the reproductive years [8]. Pregnancy in itself has been rated as a potentially stressful life event [9,10] and, given that the antenatal and perinatal period involves frequent contact with the healthcare system, this period may be experienced as particularly stressful by pregnant women who have had previous experiences of AHC, carrying the potential to affect reproductive health.

To prevent the continuation of AHC, and to support and optimise the health of women who have experienced AHC and who are pregnant or in labour, we need to improve our understanding of what qualifies as an experience of AHC and how women with such experiences manage pregnancy and childbirth. The aim of this study was therefore to describe how women with previous experiences of AHC gave meaning to and managed their experience during pregnancy and childbirth.

## Methods

### Research design

In accordance with the objective of the study, i.e. to describe how women with previous experiences of AHC gave meaning to a managed pregnancy and childbirth we applied a qualitative design in that we were interested in thick descriptions of the phenomenon [11]. Systematic Text Condensation (STC) inspired by the psychological phenomenological method described by Giorgi and modified by Malterud [12] was used for analysis. The present study was conducted as a sub-study of the Danish part of the BIDENS-Study (acronym of Belgium, Iceland, Denmark, Estonia, Norway and Sweden), which was performed between 2008 and 2010 to investigate associations between negative life events and various birth outcomes among an unselected cohort of pregnant women. At the Danish consent form for the BIDENS-Study the women were asked if the researchers were allowed to contact them for further research purpose. Estimated, more than 95% of the women gave their permission for this.

### Sampling strategy

**Figure 1:**
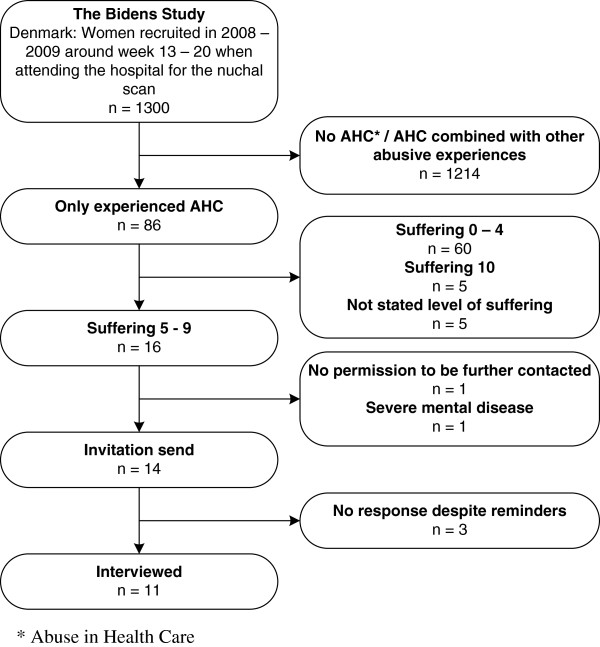
Flowchart of participant selection based on purposeful sampling.

The self-administered comprehensive questionnaire used in the BIDENS-study comprised the validated NorAQ questionnaire [1], which included three questions to assess AHC, followed by an 11 point scale to state the level of suffering due to the experience of abuse. For the present study women were invited to participate after having given birth, though based on the responses to the questionnaire which they completed during pregnancy:

Participants were selected by means of purposeful sampling [13], using a combination of criterion- and intensity sampling [13]. They were considered eligible if they 1) had responded positively to at least one item of AHC in the BIDENS-Study (i.e. experienced mild, moderate or severe AHC) whether experienced in childhood or in adulthood (Table 1), 2) had not indicated experiences of other types of abuse (to obtain as pure a description of the phenomenon as possible), and 3) scored 5–9 on a 0 – 10 point scale included in the NorAQ, measuring amount of suffering as a consequence of the experience of abuse (Table 1). The intensity sampling strategy was applied to obtain rich examples of the phenomenon, but with the intention of avoiding extreme cases that could potentially distort the manifestation of the phenomenon [13]. We therefore excluded women who ranked highest (suffering equivalent to 10) on the suffering scale. Further exclusion criteria were perinatal loss of an infant and severe mental disease, based on hospital records. Figure 1 shows a flowchart of participant selection.

**Table 1:** The items relating to abuse in health care used in the Bidens cohort study

**The following questions deal with abuse in the health care system. We ask you to mark whether you have experienced any of the following events; as a child or as an adult. If you answer yes to any of the questions we conclude – in this study – that you have been subject to abuse in the healthcare system**												
(defined as *mild abuse*)	Have you ever felt offended or grossly degraded while visiting health services, felt that someone put pressure on you or did not show respect for your opinion – in such a way that you were later disturbed by or suffered from the experience?											
(defined as *moderate abuse*)	Have you ever experienced that a“normal” event while visiting health services suddenly became a really terribly unpleasant experience, without you fully knowing how this could happen?											
(defined as *severe abuse*)	Have you experienced anybody in the health services purposely – as you understood it – hurting you physically or mentally, grossly violating you or using your body or your dependent relationship for his/her own purpose?											
	**Answer alternatives for all items:**											
	1 = No											2 = Yes, as a child (younger than 18)
	3 = Yes, as an adult (18 or older)											4 = Yes, both as child and adult
**How much are you suffering now from the consequences of the abuse in health services you have experienced?** answer by marking the number that best corresponds to how much you are suffering now												
**Don’t suffer at all**	**□**	**□**	**□**	**□**	**□**	**□**	**□**	**□**	**□**	**□**	**□**	**Suffer terribly**
	**0**	**1**	**2**	**3**	**4**	**5**	**6**	**7**	**8**	**9**	**10**	

These items were used to select participants for the present study. However, the level of severity was not given in the original questionnaire.

### Procedure

An invitation letter was sent to the 14 selected women stating the purpose of the interview and explaining that the invitation was due to their experience of AHC. The women were encouraged to contact the first author; otherwise they were contacted by telephone two weeks after the invitation was sent. Shortly before the interview the individual participants were sent a letter containing an overview of the questions.

### Semi-structured, in-depth, individual interviews

Data were collected by means of semi-structured, in-depth, individual interviews.

An interview guide was developed (Table 2) based on the aim of the study, on the current literature on AHC [1,2,4,5,7,14] and on other topics addressing similar aspects [15-17]. To structure the interview guide we were guided by the salutogenetic perspective by Antonovsky [18], which focuses on why people remain healthy despite the omnipresent existence of stressors in human existence as described by Hollnagel et al. [19]. It contains the concept of Sense of Coherence (SOC), consisting of the three components: comprehensibility, manageability and meaningfulness [18]. The women were encouraged to speak freely of their experiences, and the interview guide was solely used as a backup for the interviewer.

**Table 2:** An overview of the interview guide

**Focus area**	**Main questions (selected)**
**Comprehensibility**	Could you please try to describe the situation that led you to answer the questionnaire the way you did?
	Why do you think the abusive act happened?
	How did you experience your meeting with the healthcare system before the delivery? During the delivery? After the delivery?
	What do you think of the concept of AHC?
**Manageability**	How did you manage the abusive situation, both during it and afterwards?
	What do you think is the best way AHC can be avoided?
**Meaningfulness**	How did it affect you afterwards?
	How did you imagine your delivery in relation to your experience?
	How has the experience affected your delivery?
	How are you affected by the experience today?

The interviews were undertaken in a preferred place chosen by the woman: one in a hospital office, one in a hotel foyer, and the rest in the women’s homes. In some interviews the baby was present, which necessitated extra time for the interview. The interviews lasted between 53 minutes and 2 hours, with an average length of 1 hour and 25 minutes.

### Data analysis

The interviews were recorded digitally and transcribed verbatim. To analyze the transcribed interviews, a data-driven analytical style, described as ‘editing organizing style’ by Crabtree and Miller [20], was applied using STC [12]. A three-step analysis procedure formed the basis of the analysis: 1) In order to obtain a general and comprehensive impression of the gathered information, we listened to and read the material through thoroughly several times, bracketing own preconceptions [12] such as the salutogenetic perspective. Once we had obtained a sense of the whole, we identified ‘meaning units’, which are units of the text providing knowledge of the phenomenon being studied [12]. This was ensured by carefully searching for different key terms and expressions that appeared to *express a self-contained meaning from a psychological perspective*[21] (p 53) to the experience of the abuse and its consequences. 2) We then abstracted the content of the individual meaning units into particularly revealing themes. 3) The themes were subsequently decontextualised and further condensed into more general and abstract categories. To minimise the effects of the researchers’ individual preconceptions on the analysis, the investigators met several times to discuss and rewrite the codes and categories. The analytical process is illustrated in Table 3.

**Table 3:** The analytical process

**Meaning units (selected)**	**Themes (selected)**	**Categories**
Lack of competence by healthcare professionals	The abuse as a non-physical act	Abusive acts of non-intended harm
Initial great respect for the healthcare system	Not intentional	
Complete failure by the system		
Fear of potential consequences to life and health	Not being met with empathy	Dehumanization
Fear of punishment	Not being seen as a human individual	
Being alone and abandoned	Loss of trust	
Struggling to survive		
Treated with contempt		
Fear of being seen as disgusting		
Fear of being a burden		
Not wanting to relate to bodily femininity	Distorted perception of body	Bodily remembrance
Lost confidence in capability of body	Affected reproductive health	
To experience severe fear of childbirth		
Future wish for CS		
Rather adopt than go through childbirth		
Creating a sense of security	Finding inner strength	Finding the strength to move on
Confronting own fear	Getting help from others	
Learned to trust one’s self		
Finding the strength when crossing barriers		

### Ethical considerations

The women were assured confidentiality and written informed consent for participation in the study was obtained from all the women. Arrangements were made to ensure referral to a psychologist at the hospital if necessary. The women were informed of this possibility at the end of the interviews, but none of them made use of this opportunity.

Under Danish law, ethical approval for non-invasive studies is not required. However, due to the sensitive issues involved, we presented the survey to the Research Ethics Committee of Copenhagen, who found no objections to the study (H-1-2010-FSP4).

## Results

### Description of participants

Characteristics of the 11 participants are presented in Table 4. The mean age of the women was 32 years and approximately half of them were multiparous. The women had given birth between 7 and 19 months prior to the interviews. The women with a self-reported moderate or severe experience of abuse scored highly on the suffering scale (8 and 9, respectively). However, some of the women with a combined mild and moderate experience of AHC also reported suffering in the high end of the scale (data not shown).

**Table 4:** Participant characteristics (n = 11)

**Characteristics**	**Frequency**
**Age,** age (min – max)	32 (26 – 43)
**Marital status**	
Living with the father of the baby	10
In a new relationship	1
**Years from interview since initial experience of abuse, mean (min – max)**	7 (2 – 18)
**Grading of abuse according to questionnaire**	
Mild	3 (27%)
Mild + moderate	6 (56%)
Moderate	1 (9%)
Severe	1 (9%)
**Suffering at time of answering questionnaire (scale range 0 – 10)**	
5	4 (37%)
6	1 (9%)
7	2 (18%)
8	2 (18%)
9	2 (18%)
**Parity**	
Primiparous	5 (44%)
Multiparous	6 (56%)
**Educational level, above secondary school (12 years)**	
3 – 4 years	6 (55%)
Student at university level	1 (9%)
University	4 (36%)

### Systematic text condensation

The analytical process revealed four major categories describing the phenomenon: *abusive acts of non-intended harm*, *dehumanisation, bodily remembrance* and *finding the strength to move on.*

### Abusive acts of non-intended harm

The participants all clearly remembered the experiences categorised as AHC in the questionnaire. For two of the women, the events were related to childhood experiences of gynecological examinations due to severe pain. The events for the remaining nine women were all related to non-physical acts. For some women the event had occurred in relation to a previous pregnancy or delivery and for others it was experienced when using the healthcare system under other circumstances (i.e. in relation to an induced abortion, and during examinations for what the woman feared to be cancer). Several women described subsequent experiences of AHC after their initial experience.

Most of the women described having an initial perception of the concept of AHC as an intended act of physical and/or sexual harm and they expressed uncertainty as to whether they had been exposed to AHC at all.

Well, I think it [AHC] is such an overwhelming concept, but that is because I relate it to rape or abuse in childhood or… I know it can be used also in my situation, but it just seems so very powerful. But somehow also what one goes through. (Interview 3, experienced AHC during gynecological examination as a teenager).

However, a woman also spoke of her experience as being: “the opposite of abuse; an experience of totally neglect”. Another woman emphasised that victimisation is often considered a consequence of abuse, and that she did not want to think of herself as a victim. However, after telling their stories, most of the women stated that their experiences could be classified as having suffered an act of abuse.

“My thought was:’ Wow, this [AHC] I have never experienced. What an awkward thing to ask me about!’ But when I read the description of the concept, I thought differently. When you started talking about it… then I also came to think of how I am affected today by just a single episode in the healthcare system” (Interview 5, experienced great insensitivity in relation to a lump, which she feared to be cancer).

At the same time, the women emphasised that they did not think the act was necessarily carried out with the intention to harm them.

### Dehumanisation

The abusive acts were accompanied by feelings that shattered the women’s self-perception.

#### Violated trust

The participants repeatedly described how they had firm belief in the medical authorities before the abusive act. This feeling originated from a deep respect of the medical disciplines and the knowledge and skill involved therein. This trust in the medical authorities was by some of the women further emphasized as the main reason why they deliberately chose to, and were able to, cede sovereignty over their bodies. The feeling of abuse occurred when the women felt that their trust was violated, for example by insufficient expertise of the healthcare professionals treating them e.g. medical doctors, nurses or midwives. This violation of trust led to mistrust of the abusive healthcare worker in particular, but to some women the mistrust extended to the whole of that profession.

Although a firm belief in the medical authorities was evident throughout most of the interviews, one woman expressed that her experience was not related to this aspect. Instead, she explained that it could be attributed to institutional abuse due to general failings of the organisation, such as insufficient time, budget cuts affecting health care, disrespectful prioritisation, and a sense of being treated with boundless powerlessness, all leading to a distrust of the whole healthcare system.

The women described an inability to recognise themselves in the abusive situation. They usually perceived themselves as autonomous, well-educated, independent women. However, their trust in medical authorities weakened their independence and autonomy and made them more vulnerable.

“Well I think of myself as a very independent person capable of making a plan and then sticking to it. And before I came here [at the hospital] I had ideas [of how to manage the situation]. But upon arriving I could not remember these ideas. They had been pushed aside by the authority that the hospital represented” (Interview 1, had felt ignored and abandoned during an examination at the delivery ward).

Some women described how they feared that the medical staff would punish them by refraining from giving them adequate treatment if they objected to the way they were treated.

“It is difficult, because somehow you are in the hands of those treating you. I know that you are not supposed to say so… it is not meant to be so… that you get punished if you say something” (Interview 4, had been given the impression that her life was in danger and that proper treatment was then withheld due to controversies among the attending staff).

During the abusive act, it was this feeling that stopped them from acting upon their experience.

Thus, the feeling of violated trust caused an internal struggle between the women’s acceptance of authority and their need to acknowledge their independence and self-respect.

#### Not being embraced as an individual human being

The feelings of abuse arose in situations where the women experienced that the hospital staff did not treat or respect them as individual human beings. Lack of interest in the individual made the women experience externally imposed attributes (e.g. fear of childbirth, being a loser, or being irresponsible and stupid), which they had not previously felt, and this amplified their sense of AHC. This indifference also caused feelings of being ignored, deeply humiliated and worthless.

“I think it is very much human nature that you want to be informed … you become like an animal if you are examined without anybody telling you anything” (Interview 5).

Further, all the women mentioned lack of empathy as a substantial part of their experience of AHC.

I personally think it was pretty harsh that I was tied down [during vaginal examination]… and I was so scared. He [the doctor] did not pay any attention and was cold as ice. Terrible cold. He did not pay attention to the fact that I was a child. Not even a teenager but a small child (Interview 11, experienced AHC during gynecological examination as a child).

Some women had feared for their own life or for the life of their babies. However, it was the accompanying sensation of being subject to insensitivity, arrogance, sloppy work, or even witnessing conflicts among the attending healthcare professionals that led them to report AHC in the questionnaire.

My life was somehow in their hands. And then hearing someone saying:‘ We do not have the time’ At that point all I was told was that… they thought it was an artery that had been torn, so I knew it was serious. In my mind that meant: ‘Well, there is no time, you will bleed to death’ (Interview 4, overhearing the delivering obstetrician arguing with the anesthetist over the prioritization of the operating theatre).

Despite the gravity of their experiences, the women did not dare or did not feel capable of acting in response to these situations, or had simply not managed to make the staff aware of their fears, despite several attempts to do so.

The experience of ignorance made the women feel abandoned, lost, insecure and let down in situations where they had initially expected to receive support. They therefore felt forced into making decisions about matters that could not only potentially result in their own death, but could also put the lives and safety of their children at risk. They felt that the heavy burden of these decisions had been forced upon them alone.

For some of the women, the abuse also had an impact on their perception of their ability as mothers. This was evident both indirectly, through feelings of frustration when they were unable to feed the baby sufficiently, and more directly e.g. when the healthcare personnel questioned whether the mother suffered from postnatal depression. One woman also described how the experience of AHC had negatively affected her initial attachment to her baby.

The feeling of being a burden to the healthcare system was also mentioned. The women often delayed or refrained from contacting the system until they felt it was unavoidable. In doing their utmost to gain knowledge of their potential disease and only contact the health care system when it was absolutely necessarily, they felt even more humiliated and confirmed in their sense of being a burden when they were treated with neglect and insensitivity.

### Bodily remembrance

The abusive experiences also resulted in long-lasting consequences for the women.

#### Bodily consequences

For many of the women, the abuse had far-reaching consequences for the perception of and respect for their own bodies. For some, it became difficult to accept their bodily femininity and resulted in difficulties relating to their bodies as well as problems in sexual relationships.

“… And it took me several years to get a proper understanding of my own femininity. I mean; how to relate to my own body and to sex …and…I do not want to blame the exact treatment I was given in relation to the abortion, but it certainly played a part in me being confused … in how to relate to my body and how to be respected with my body.[…] I have had a fairly detached relationship with my body and my sexuality” (Interview 6, experienced AHC in relation to a termination of pregnancy as a teenager).

The bodily consequences also manifested themselves in daily life in other ways, e.g. as panic attacks and were experienced regardless of whether or not the situation of abuse was related to a physical act.

#### Reproductive implications

The experiences of abuse also lead to grave concerns about getting pregnant and giving birth. The women who had experienced abuse at an early stage in their lives spoke of how they had always imagined themselves adopting children rather than going through pregnancy and childbirth. They explicitly expressed how their experiences led them to suffer from a severe fear of childbirth. However, for one of the women the desire to have children outweighed the fear, and another got pregnant unplanned; “to my own luck…” as she expressed. Other women described how the experiences of AHC led them to imagine or actually plan delivery by caesarian section rather than by giving birth vaginally.

“… I have to be almost certain that I can have a cesarean if I am to consider getting pregnant at all [again]. If I cannot have a cesarean, then I am not having any more children” (Interview 1).

Alternatively, one woman described how she managed her fear of childbirth by simply refraining from thinking of the delivery.

Experiences in adulthood also affected the decision, regardless of a continuing desire, to have (more) children: one woman, who had experienced severe degradation and humiliation during her first delivery explained how she could no longer pass the hospital in her everyday life without starting to cry. It took her three years to even dare getting pregnant again.

The women described how they thought their experience of AHC had lead to complicated deliveries, in which they felt abused by the healthcare system all over again.

The experience of AHC not only affected the women themselves but was also perceived to influence the children. One woman described how her baby’s restlessness, colic and difficulties being in large crowds could have been caused by her experience of AHC.

### Finding the strength to move on

The women had found different ways of managing their experiences of AHC.

#### Finding inner strength

When dealing with their experiences of abuse the women often spoke of the feeling of an inner strength, which gave them confidence to act in renewed meeting with the health care system, and to believe in themselves again. One effective strategy to achieve understanding and respect, and to ensure that the experience was taken into consideration, was to tell the healthcare professionals of the experience of AHC.

It was also emphasised that the pregnancy and delivery itself had relieved some women from some of the distress they had experienced. They felt their bodies had gained new meaning and purpose, which made them feel comfortable and strong.

“Being pregnant… was to me somehow an ‘aha’ experience… like ‘well this is what my body is meant for, this is what my gender is also capable of.’ I am a lot of other things and my intellect is a lot of other things other than my body; ‘But well, this is how it is being a woman. And wow, imagine that I am capable of that” (Interview 6).

Furthermore, motherhood reduced the severity of their experience of AHC, making it fade into the background. However, becoming a mother had also forced some of the women to struggle to be seen, heard and respected not only for themselves but now also for the sake of their child.

Finding this inner strength was not necessarily seen as a purely positive thing. Some women explained that although they had become aware of their inner strength, they would still rather not have experienced the abuse.

#### Finding strength through others

Several of the women had to seek help from a psychologist due to their experience of AHC and felt supported by this action and the help they received. When asked what could relieve them from some of the distress caused by the experience, many women explicitly spoke of situations in which they had had positive encounters with healthcare professionals, and they emphasised how this had a great potential to give them the strength to move on.

“I am a more positive person now because I have had a good experience and have found out things… I have had a really positive experience at the delivery ward. If I had not had that, then I simply don’t know… I suppose it would have gone completely wrong” (Interview 11).

Repeatedly, the women stated that they felt heard and understood when their concerns were met with empathy and genuine interest. The healthcare professionals could achieve this in various ways such as knowing the patient’s name, giving them their undivided attention, displaying knowledge of the hospital records, and making eye contact. The women also emphasised the importance of sufficient information that was tailored to the individual, and how ensuring continuity created a sense of safety and of feeling respected. The importance of feeling in control over their own body was also frequently mentioned. When the healthcare professionals managed to support the women in feeling this way, the women felt strengthened and less frightened. Further, they felt more confident when they were treated by a healthcare professional they experienced as skilled, knowledgeable and empathic.

*I got a midwifery student who simply… made sure that he was born. It was thanks to her that he was born that way [vaginally]. She was fantastic, simply magnificent. She really calmed me down, she was so gentle … really took care of me and told me everything she did and what I was supposed to do. I was constantly in contact with her.****Interviewer: What did you need in particular?****I needed her, she did everything I needed. I had explained my story to her and that I felt it was very difficult. And she accepted that and responded to my needs (Interview 11).*

When asked, the women generally stated that healthcare personnel should not be afraid to enquire about previous experiences of abuse. However, as one woman emphasised, it would require a feeling of confidentiality in an established atmosphere of continuity in order to feel comfortable enough to answer.

## Discussion

We explored how women with previous experiences of AHC comprehended, managed and made sense of their experience of AHC during pregnancy and childbirth, and found that the phenomenon could be described by four categories. We found AHC to be understood as *acts of unintentional harm*. However, controversy in the literature exists as to the intentionality of AHC. In a charter on respectful maternity care seven major categories of disrespect and abuse during maternity care were described, ranging in a continuum from subtle disrespect and humiliation to overt physical abuse [6]. In our study AHC is defined by the NorAQ questionnaire and encompasses both subtle types of abuse and potential overt physical, mental or sexual abuse. The women in our study had mostly experienced abuse that could be classified as neglect or verbal abuse, which are often more subtle types of abuse. However, the importance of experiencing subtle types of abuse must not be ignored, as neglect has been described as the most distressing aspect of AHC [5].

Based on reports mainly from South America and Africa, d’Oliveira [5] stated that violent abuse is often deliberate, whereas Brüggermann et al. [3] recently reported that the events are most often unintended, which is in line with our findings. The discrepancy with regard to intentionality of AHC is likely to reflect cultural and ethical differences in the provision of healthcare. AHC is a relatively new research area [14] and has only recently been operationalised as a phenomenon that *‘has severe consequences but is invisible if seen from a medical error or a patient satisfaction perspective’*[3] (p 1). Our findings support this definition, as the abusive acts were only rarely related to medical errors. Instead, they resembled the feeling of devoid of care, as described by Brüggermann et al. [3].

We found *dehumanisation* to constitute a main category of AHC. This finding is in agreement with Haslam [22], stating that dehumanisation features prominently in writings of modern medicine *‘…with its lack of personal care and emotional support’*[22] (p 253). Haslam described animalistic dehumanization, which is often accompanied by degradation and humiliation by equating individuals with animals. It often carries a prominent bodily component featuring disgust and revulsion of the dehumanised others [22]. We found that whatever the intent, the experience of AHC resulted in long-lasting psychosomatic consequences in some women, implying that this bodily component not only exists as a means of dehumanisation, but also severely affects the dehumanised person. Haslam also describes mechanistic dehumanisation, which is characterised by indifference and lack of empathy [22]*.* Our participants described feelings of being completely ignored, not being met with empathy, and not being respected as an individual human being, which - according to Haslam - are encompassed in mechanistic dehumanization.

Haslam discusses the presence of empathy as a requirement for overcoming dehumanisation [22]. The importance of empathy is increasingly recognized in the medical professions [23]. However, the medical definition differs from the use of the term outside the field of medicine: the clinical definition is purely cognitive, contrasting it with sympathy [23]. Thereby the experience of AHC could be intensified, if the healthcare professionals think they are showing empathy but this is not perceived as such by the patients.

A qualitative study among gynecological patients found that AHC was explained by the core category of being nullified [14]. Our category of dehumanisation has aspects that overlap with nullification. However, we also found that violated trust was a theme within dehumanisation. Trust is believed to be essential for effective therapeutic encounters [24] and therefore violation of trust has significance for both the patient and the healthcare system. Hall et al. [24] state that violation of trust tends to produce an emotional reaction of outrage or indignation rather than merely disappointment, which explains the serious consequences seen among our study participants.

Previous experience of AHC compelled the women to delay or refrain from contacting the healthcare system. In support of this finding, fear of embarrassment due to concerns about being labeled as neurotics, hypochondriacs, or time-wasters has also been recognised as an important barrier to seeking help in cancer patients [25].

We found that the category of *bodily remembrance* included the impact of AHC on the reproductive health; this manifested itself as reluctance to/fear of getting pregnant or giving birth vaginally, and in the initial mother-infant relationship. Fear of childbirth was significant in some women, and one woman dealt with this by repressing all thought of the upcoming delivery; she also experienced the delivery as retraumatising. This implies that the experience of AHC can lead to anxiety accompanied by avoidance behaviour, with the potential of retraumatisation.

For the multiparous women AHC was related to previous negative obstetrical experiences. The literature supports the finding of obstetrical disrespect and abuse [4,5,17,26]. Further, in accordance with another study [27] we found that previous birth experiences did not cause distress because of the mode of delivery, but because of the inter-personal relationship with the caregiver. These experiences might influence the choice of mode of delivery in future pregnancies, leading to a rise in cesarean sections on maternal request, which could potentially increase obstetrical complications [28].

The category of *finding the strength to move on* described how the women dealt with their experiences. The women spoke of pregnancy and childbirth as a period in their lives that had the potential to give them strength despite their experience of AHC. Healthcare personnel could contribute to this process by showing genuine interest and empathy.

### Methodological considerations

We aimed to deepen the understanding of the complex phenomenon of AHC by using systematic text condensation (STC) according to Malterud [12]. STC is based on phenomenology, which seeks to describe the meaning of lived experiences [20,29], not as reality is specified in advance, but as it is perceived by the participants [30]. Consequently, care has to be taken to bracket the researcher’s preconceptions. We had expected the women’s experience of AHC to directly influence pregnancy and childbirth. However, we also discovered that the experiences were embedded in their whole life situation and by this also affected their reproductive lives.

When conducting the interviews and the analyses we also carefully tried to bracket the background of our professions (midwifery and psychology). As such, it was of importance not to feel personally responsible for the women’s experiences, but at least as important not to trivialize the meaning of the women’s narratives. Thus care should be taken not to evaluate their experience by the ‘objective’ severity rather than by their stated suffering: experiences of mild/moderate AHC also caused substantial distress, as reflected by the high level of suffering.

Trustworthiness was ensured by researcher triangulation. The authors represented two different disciplines, which gave rise to different perspectives of the research field, and which served to strengthen validity [31].

We acknowledge the seeming dilemma inherited in the study; phenomenological studies are by definition data-driven whereby one should refrain from projecting own theories. Contrary to a pure phenomenological approach, in STC theoretial perspectives are applied moving between identification with or bracketing a specific perspective during the different steps of the analysis process [32]. Although this study was initially guided by the theoretical concept of salutogenesis, the analysis remained entirely datadriven and Antonovsky’s theory was not used as a template, but solely helped to define the research question and to structure the themes in the applied interview guide. Thus the aim was not to validate the salutegonetic model proposed by Antonovsky but instead allow new meaning to emerge based on what the participants thought and felt was relevant in relation to the chosen phenomenon.

It is our impression that it took some women a relatively long time to gain the confidentiality to speak freely of their experience. In this regard, one woman explained that she felt reluctant to speak to the interviewer, because the researcher came from the same professional background as the healthcare professional she felt abused by. However, the women also spoke of how they felt it had helped them to tell their stories. According to McCoyed et al. [33] the interview process may possess therapeutic benefits to the research participants and lead to an experience of social validation, if feeling that the story is accepted. In this respect, our findings could also reflect the effects of a therapeutic talk, which may have provoked a level of reflectivity in the participants that could influence the description of the lived experience.

An ambiguity in the concept of AHC was revealed by our study, as many of the women did not initially consider themselves to have experienced abuse. Despite this belief, the experiences had substantial impacts on the women, and in some resembled the consequences of other forms of abuse [34,35].

We acknowledge that the study has several limitations. Being a qualitative study the women were purposefully sampled, among others based on no indications of other types of abusive experiences. Even though physical, sexual and emotional abuse occurs among all social classes, younger age and lower education has been associated with a higher prevalence of abuse [36]. As a potential result of the sampling procedure the women in our study were all well educated, thereby limiting the transferability of the results. Further, the women were sampled as to their level of suffering as stated in the midst of their pregnancies. Due to the vulnerability that this period also represents, pregnancy itself might have led the women to score higher on the suffering scale, limiting the transferability beyond the study setting.

Also the choice of using STC when analyzing the data brings limitations to the study. STC can be considered a cross-case method [32]. Hereby the longitudinal aspect of the individuals’ narratives carries the risk of being lost [32]. However, we believe that focusing also on how the women managed their experiences has limited the side effects of the cross-case design.

However, in accordance with existing literature, our findings describe how AHC affects the lives - and in particular the reproductive lives - of the abused women. This combined with the description of the experience and recommendations on how to deal with it may provide important theoretical knowledge to healthcare staff in situations other than those outlined in this study.

Halpern [23] states that empathy facilitates trust and can be directly therapeutic, which is why we believe it would be of clinical value to encourage women who have experienced AHC to tell their story and thereby potentially help them overcome their experiences. For this process to be effective, it is important to acknowledge without victimising women with experiences of AHC.

## Conclusion

In the cultural context in which the study was undertaken experiences of AHC were often considered as unintended acts of harm arising from both physical and non-physical acts. Nevertheless, the study findings suggest that regardless of whether AHC was experienced in childhood or adulthood it could have substantial consequences, leading to feelings of dehumanisation and potentially further leading to distorted body perceptions and to affected reproductive health. However, the study participants also revealed potential resources that were important for them in order to confront, comprehend, and manage their experiences. When addressing future strategies for avoiding AHC it is important to acknowledge the various forms of dehumanisation [22], focusing on the importance of its opposite: empathy.

## Abbreviations

AHC: Abuse in Health Care; BIDENS: Acronyms of Belgium, Iceland, Denmark, Estonia, Norway and Sweden six countries participating in a multinational cohort study in which the women were sampled; NorAQ: NorVold Abuse Questionnaire; SOC: Sense of Coherence

## Competing interests

The authors declare that they have no competing interests

## Authors’ contributions

AMS and JM planned the study and developed the interview guide. The interviews were conducted by AMS. AMS, HK and JM all contributed to the analysis. AMS drafted the initial manuscript and all authors contributed to the final version. All authors read and approved the final manuscript.

## Authors’ information

On behalf of

The Bidens Study Group, who undertook the multinational part of the Bidens Study: Berit Schei (principal investigator), University of Trondheim, Norway; Elsa Lena Ryding (co-principal investigator), Karolinska University Hospital, Sweden; Mirjam Lukasse (coordinator), University of Tromsø, Norway. Local principal investigators: Marleen Temmerman (University of Ghent, Belgium), Thora Steingrímsdóttir (Landspitali University Hospital, Iceland), Ann Tabor (Copenhagen University Hospital, Rigshospitalet, Denmark), and Helle Karro (University Hospital Tallinn, Estonia). Local coordinators: Made Laanpere (University of Tallin), An-Sofie Van Parys (University of Ghent, Belgium), Hildur Kristjansdóttir (Iceland) and Anne-Marie Wangel (Malmö University, Sweden). The study has been supported by grants from five institutions, all mentioned in the acknowledgement section.

## Pre-publication history

The pre-publication history for this paper can be accessed here:

http://www.biomedcentral.com/1471-2393/13/74/prepub
